# Targeting of chromatin readers: a novel strategy used by the *Shigella flexneri* virulence effector OspF to reprogram transcription

**DOI:** 10.15698/mic2015.01.183

**Published:** 2014-12-28

**Authors:** Habiba Harouz, Christophe Rachez, Benoit Meijer, Christian Muchardt, Laurence Arbibe

**Affiliations:** 1 Department of Immunology, Infectiology and Hematology, Institut Necker-Enfants Malades (INEM), INSERM U1151, CNRS UMR 8253, Université Paris Descartes, Paris, France.

**Keywords:** Shigella, OspF, Heterochromatin protein 1, epigenetic, immune gene

## Abstract

* Shigella flexneri*, a gram-negative bacterium responsible of bacillary dysentery, uses multiple strategies to overcome host immune defense. We have decrypted how this bacterium manipulates host-cell chromatin binders to take control of immune gene expression. We found that OspF, an injected virulence factor previously identified as a repressor of immune gene expression, targets the chromatin reader HP1γ. Heterochromatin Protein 1 family members specifically recognize and bind histone H3 methylated at Lys 9. Although initially identified as chromatin-associated transcriptional silencers in heterochromatin, their location in euchromatin indicates an active role in gene expression. Notably, HP1γ phosphorylation at Serine 83 defines a subpopulation exclusively located to euchromatin, targeted to the site of transcriptional elongation. We showed that OspF directly interacts with HP1γ, and causes HP1 dephosphorylation, suggesting a model in which this virulence effector “uses” HP1 proteins as beacons to target and repress immune gene expression (Harouz, *et al.* EMBO J (2014)). OspF alters HP1γ phosphorylation mainly by inactivating the Erk-activated kinase MSK1, spotlighting it as a new HP1 kinase. *In vivo*, infectious stresses trigger HP1γ phosphorylation in the colon, principally in the *lamina propria* and the intestinal crypts. Several lines of evidence suggest that HP1 proteins are modified as extensively as histones, and decrypting the impact of these HP1 post-translational modifications on their transcriptional activities *in vivo* will be the next challenges to be taken up.

*Shigella* bacterial species, a causal agent of bacillary dysentery in humans, delivers through the type III secretion system (T3SS) the virulence effector OspF to directly inactivate MAPK signaling in the nucleus of infected epithelial cells. OspF inactivates MAPKs through a phosphothreonine lyase activity. This enzymatic activity irreversibly inactivates the dual-phosphorylated host MAPKs (pT-X-pY) through beta elimination of the phosphate group, converting the phosphothreonine residue required for MAPK activity into a dehydrobutyrine (Dhb). We previously showed that OspF binds host chromatin in a gene-specific manner and, by intercepting with nuclear MAPK signaling, installs repressive chromatin marks on a subset of proinflammatory chemokines, rendering chromosomal sites inaccessible to both NF-κB and the RNA polymerase II (RNAPII). Therefore, OspF acts as an epigenetic regulator reprogramming the host transcriptional response by irreversibly inactivating nuclear MAPKs, which results in an immunosuppressive environmentfavoring life of the bacterium at the mucosal surface.

**Figure 1:**
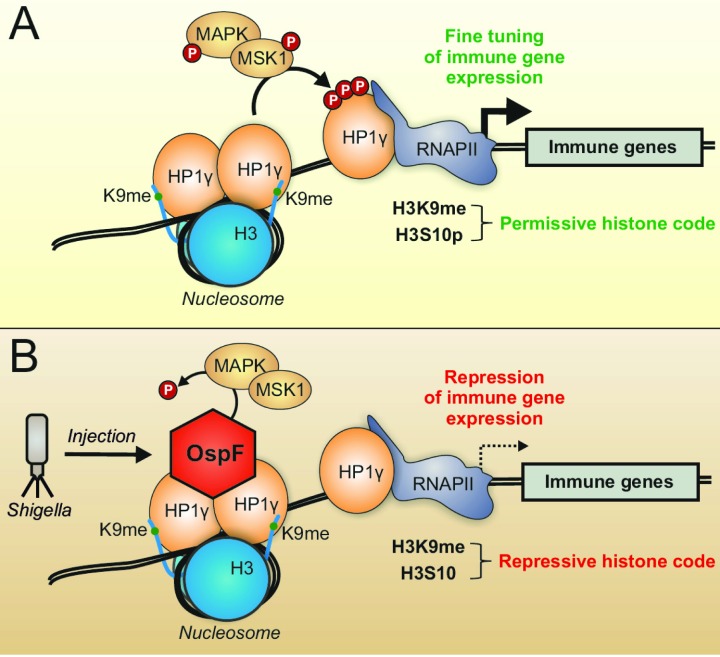
FIGURE 1: The T3SS virulence effector OspF targets HP1γ and highlights the MSK1/HP1γ chromatin interplay at immune genes. Under physiological conditions **(A)** MAPK signaling leads to HP1γ and histone 3 serine 10 (H3S10) phosphorylation, allowing fine tuning of immune gene expression. Upon infection **(B)** *Shigella *injects the virulence factor OspF into the host cell. OspF translocates to the nucleus and both, binds to HP1γ as well as inactivates MAPK irreversibly by altering the phosphorylation site. Dephosphorylated HP1γ and H3S10 lead to repressed transcription of immune genes, and thus drive *Shigella *infection.

As a chromatin-binding protein-kinase inhibitor, OspF provides an interesting tool for decrypting the interface between MAPK and chromatin. We showed that OspF directly interacts with the chromatin reader HP1γ and, once injected by the *Shigella* T3SS, modulates the HP1γ phosphorylation state in infected cells by targeting the nuclear MAPK signaling pathway. More precise decrypting of the pathway showed that MSK1, a kinase downstream of Erk, is a HP1 kinase and that OspF injection inactivates the Erk-MSK1 signaling cascade leading to HP1γ phosphorylation (Figure 1).

OspF reveals a new interplay between nuclear MAPK signaling and chromatin. Various evidences suggest that kinases interact with chromatin as integral component of transcription activation complexes at promoters and with elongating RNAPII at transcribed regions. Importantly, we identified a tight molecular complex between MSK1 and HP1γ in living cells, an interaction that possibly stabilizes MSK1 on the chromatin template, and facilitates HP1γ phosphorylation (Figure 1).

HP1 are extensively modified proteins. For example, SUMO modifications promote HP1 targeting to pericentric heterochromatin while phosphorylation at Ser 83 defines a subpopulation exclusively located to euchromatin, interacting with the elongating form of the RNAPII. At these intragenic positions, HP1γ may stabilize ongoing transcription and/or participate in both alternative splicing and co-transcriptional RNA processing, presumably by favoring the recruitment of the splicing machinery. The role of phospho-modifications on these HP1γ transcriptional activities remains poorly characterized; one report indicates that Ser 83 phosphorylation abrogates HP1γ silencing activity in a transcriptional reporter assay. Since OspF precisely targeted this Ser 83 residue, we questioned the role of HP1γ and of this phospho-event in gene expression. Using HP1γ null cell lines rescued with either WT HP1γ or with a S83A mutant, we found that upon MAPK activation, WT HP1γ was required for the activation of pathways involved in cell cycling and proliferation, purine metabolism, DNA replication and repair. These data spotlight HP1γ as a chromatin integrator for the MAPK-driven proliferative gene expression program. Conversely, at immune genes, WT HP1γ was required for fine tuning of the transcriptional activity, reducing the amplitude of the response to cell stimulation at a selective set of OspF target genes, such as CCL2, CCL20, and others. Unexpectedly, a single S83A mutation interfering with Ser 83 phosphorylation reduced but did not abolish the impact of HP1γ on transcription, suggesting the implication of additional phospho-modifications. Overall, these results suggested that HP1γ signaling promotes a transcriptional program aimed at cell proliferation and repair while dampening inflammation.

The physiological function of HP1γ remains poorly investigated since the HP1γ null mutation induces a high rate of embryonic lethality. Furthermore, only 1% of animals reach adulthood with severe hypogonadism characterized by a meiotic defect in spermatocytes and oocytes, suggesting a role in facilitating centromere clustering in early meiotic prophase. Triggering of HP1γ activation by infectious stresses remained totally unexplored. In an *in vivo* model of rectocolitis, we showed that the non-invasive - albeit proinflammatory -* mxiD Shigella *mutant promoted pronounced HP1γ phosphorylation in the colon when compared to wild type *Shigella*. PAMPs (pathogen-associated molecular patterns) such as LPS were shown to induce major dynamic changes in the cell phosphorylation state, with multiple phosphorylation sites on HP1γ, including Ser 83. Therefore, it remains possible that bacterial challenge directly -through LPS release - or indirectly initiates proinflammatory signaling cascade(s) leading to increased HP1γ phosphorylation at multiple residues, including the Ser 83 residue monitored in our study. In turn, triggering of HP1γ signaling may be an important "checkpoint" in the control of inflammatory gene activation, aimed at preventing excessive or deregulated immune gene expression (Figure 1). Thus, the development of *in vivo* approaches targeting HP1γ in the intestine will be essential to unravel these important physiological issues.

